# CdTe Nanocrystal Hetero-Junction Solar Cells with High Open Circuit Voltage Based on Sb-doped TiO_2_ Electron Acceptor Materials

**DOI:** 10.3390/nano7050101

**Published:** 2017-05-03

**Authors:** Miaozi Li, Xinyan Liu, Shiya Wen, Songwei Liu, Jingxuan Heng, Donghuan Qin, Lintao Hou, Hongbin Wu, Wei Xu, Wenbo Huang

**Affiliations:** 1 School of Materials Science and Engineering, South China University of Technology, Guangzhou 510640, China; limz4994@sina.com (M.L.); liuxy0310@sina.com (X.L.); a740176840@163.com (S.W.); majestyv@sina.com (S.L.); hengjingxuan@163.com (J.H.); 2 Institute of Polymer Optoelectronic Materials & Devices, State Key Laboratory of Luminescent Materials & Devices, South China University of Technology, Guangzhou 510640, China; hbwu@scut.ed.cn (H.W.); xuwei@scut.ed.cn (W.X.); pswbh@scut.ed.cn (W.H.); 3 Siyuan Laboratory, Guangzhou Key Laboratory of Vacuum Coating Technologies and New Energy Materials, Department of Physics, Jinan University, Guangzhou 510632, China

**Keywords:** nanocrystal, solar cells, CdTe, heterojunction

## Abstract

We propose Sb-doped TiO_2_ as electron acceptor material for depleted CdTe nanocrystal (NC) hetero-junction solar cells. Novel devices with the architecture of FTO/ZnO/Sb:TiO_2_/CdTe/Au based on CdTe NC and TiO_2_ precursor are fabricated by rational ambient solution process. By introducing TiO_2_ with dopant concentration, we are able to tailor the optoelectronic properties of NC solar cells. Our novel devices demonstrate a very high open circuit voltage of 0.74 V, which is the highest *V*_oc_ reported for any CdTe NC based solar cells. The power conversion efficiency (PCE) of solar cells increases with the increase of Sb-doped content from 1% to 3%, then decreases almost linearly with further increase of Sb content due to the recombination effect. The champion device shows *J*_sc_, *V*_oc_, FF, and PCE of 14.65 mA/cm^2^, 0.70 V, 34.44, and 3.53% respectively, which is prospective for solution processed NC solar cells with high *V*_oc_.

## 1. Introduction

Nanocrystal (NC) solar cells have received extensive attention in the last several years due to their many advantages—such as low cost, environmental friendliness, solution process, simple device technics, and compatible roll-to-roll manufacturing [1,2,3,4,5,6,7,8]. The most efficient NC based solar cells nowadays are fabricated by using CdTe or PbS NC as donors with wide bandgap semiconductor materials as acceptors [9,10,11,12,13]. Compared to PbS NC, CdTe NC is less complex and more stable in air, which permits devices to be fabricated under ambient conditions. Thus, CdTe NC thin film has been studied intensively [14,15,16,17,18,19,20]. The working principle of efficient CdTe NC solar cells is based on the p-n heterojunction that contains two media: an electron donor and electron acceptor. To prepare a high quality CdTe NC donor absorber, a layer by layer sintering process should be carried out to eliminate stress and defects in CdTe NC thin film [21,22]. The electron acceptor materials—typically ZnO, CdSe or other n-type semiconductor thin films—are thus the key to improve the device performance. The most efficient CdTe NC–ZnO solar cells are fabricated by using a normal structure of ITO/CdTe/ZnO/Al [10]. In this device architecture, Zn^2+^ precursor is deposited on the CdCl_2_ treated CdTe NC thin film and annealed at a moderate temperature of 300 °C, which enables high quality junction formation and avoids large current leaks. However, efforts try to duplicate this device with an inverted structure of ITO/ZnO/CdTe/Au failed due to the poor junction quality in the ZnO–CdTe interface, which had been confirmed in our previous work [23]. It is noted that there are many merits for solar cells with inverted structure, such as a charge separating interface close to the illumination and usage of metal or metal oxide with high work function as a hole-collecting electrode, which endows device long lifetime. Solution processed CdTe NC based solar cells with inverted structure of ITO/CdSe/CdTe/Au were reported for the first time by Towsend et al. [19]. Although as high as 3.8% PCE was obtained in the above devices, they suffered from low *V*_oc_ (< 0.5 V) value, much lower than that (up to 0.8 V) of CdTe/CdS devices prepared by close space sublimation (CSS) method [24]. With Cr/Au as hole collecting electrode, the *V*_oc_ of devices with the structure of ITO/CdSe/CdTe/Cr/Au can be further increased to 0.62 V [20]. By using ZnO as the interlayer, devices with an architecture of ITO/ZnO/CdSe/CdTe/Au obtain a high *V*_oc_ of up to 0.6 V in conjunction with a high PCE of ~6% reported in our recent work [25,26]. Recently, Yang et al. [27,28] developed an in situ route to construct CdTe–CdS NC bulk heterostructure solar cells by direct thermal treatment of mercaptoethylamine stabilized CdTe NC. In this device structure, the formation of n-CdS shells on CdTe NC eliminated the recombination of carrier and improved the device performance. Recently, [29] introduced p type spiro-OMeTAD as the hole transport layer between CdTe NC thin film and Au electrode, as high as 0.71 V of *V*_oc_ was obtained, which was the highest *V*_oc_ value ever reported for solution-processed CdTe NC based solar cells. Another way to improve the *V*_oc_ of CdTe NC based solar cells is to engineer the bandgap structure of the photo-generated electron-accepting materials. As previously reported [12], the trap state density of CdTe was 10^14^ cm^−3^, high doped density of n-type materials was necessary to fully deplete the CdTe NC film in order to increase the carriers’ separating and collecting efficiencies. It had been found that, by using n type doped TiO_2_ as the electron acceptor, the performance of PbS colloidal quantum dot/TiO_2_ heterojunction solar cells can be tailored and improved substantially [30]. Bulk hetero-junction structure was built as porous structure formed during the decomposition of Ti-sol, which improved carrier collecting efficiency. Herein, we introduce solution-processed CdTe NC/TiO_2_ hetero-junction solar cells consisting of ZnO film, thermal decomposition Sb-doped TiO_2_ film, and solution processed CdTe NC thin film. The carrier separating/collecting efficiency of solar cells can be improved by introducing Sb-doped in TiO_2_ as the electron acceptor. It is found that *V*_oc_ up to 0.7 V can be obtained with a Sb-doped TiO_2_ electrode and as high as 0.74 V is obtained in the case of 8% Sb-doped TiO_2_ devices, which is the highest *V*_oc_ ever reported for solution-processed CdTe NC based solar cells.

## 2. Results and Discussion

**Figure 1:**
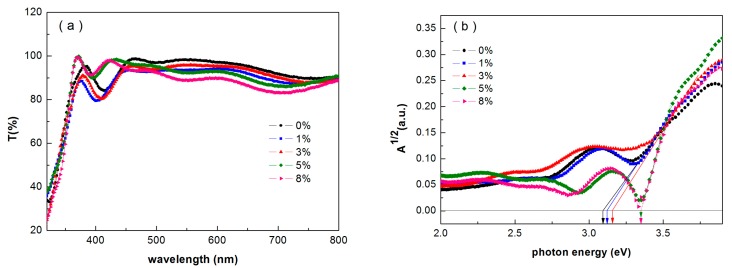
(**a**) Transmission spectra and (**b**) optical absorption curves of Sb-doped TiO_2_ thin film.

In the case of inverted structure CdTe NC based solar cells, the absorbance or transmission of window layers has a great effect on the light passing through the CdTe NC active layer. Window layers with a high light transmission in short wavelengths will increase the response of spectrum in the device. Figure 1 shows the transmission spectra and the root mean square of the absorption against photon energy of Sb-doped TiO_2_. The measurement is taken by using FTO as the standard sample. From Figure 1a, it can be seen that the FTO/ZnO/Sb-TiO_2_ thin films block the light with a wavelength shorter than 400 nm and show almost transparent behavior for wavelengths from 400 to 1000 nm. A little decrease in the transmission is found when the Sb doping concentration increases to 8%. As shown in Figure 1b, the bandgap of the Sb-doped TiO_2_ layer utilized in this work is in the range of 3.1–3.3 eV, which is derived by taking a tangent of straight line of this curve at *A* = 0 (*A* = (*αhν*)). There are almost no changes with different Sb doping ratios, which imply that Sb doping does not change the bandgap of TiO_2_ thin film, in accordance with the previous report [30].

**Figure 2:**
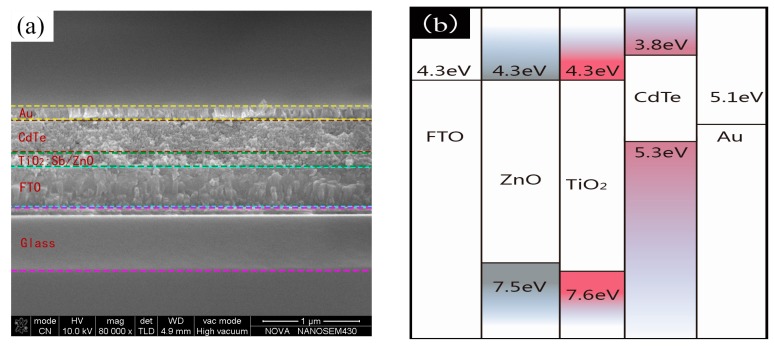
(**a**) Cross-sectional SEM images of the FTO/ZnO/TiO_2_/CdTe/Au device architecture; (**b**) band alignment of FTO, ZnO, TiO_2_, CdTe, and Au.

To build solar cells devices, we deposited five layers of CdTe NC thin film on 50 nm Sb-doped TiO_2_ and 40 nm-thick ZnO thin film. Au is deposited on the CdTe layer to make ohmic contact. The cross-sectional SEM micrograph of this CdTe–TiO_2_ device is also depicted in Figure 2a. The CdTe NC film is homogeneous with thickness ~400 nm. The band alignment of solar cells is presented in Figure 2b. Photoelectrons are generated in the CdTe NC active layer and separated in the CdTe/TiO_2_ interface. The electrons are transported to ZnO and collected by the FTO, while photogenerated holes traveled to the Au electrode.

**Figure 3:**
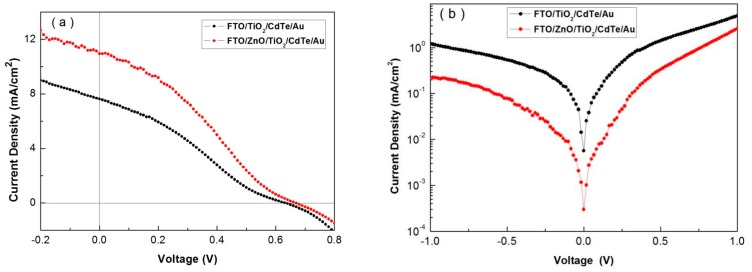
*J*–*V* characteristics of FTO/ZnO/TiO_2_/CdTe/Au and FTO/TiO_2_/CdTe/Au devices (**a**) under light and (**b**) dark.

The presented ZnO thin film can provide better electrical stability and the smooth and pin-hole free TiO_2_ film can be grown on it, which is confirmed in our previous work [25,26]. Furthermore, it can eliminate catastrophic shorts from the upper contact directly through CdTe layers to the FTO. For comparison, devices with structures of FTO/ZnO/TiO_2_/CdTe/Au and FTO/TiO_2_/CdTe/Au are fabricated under the same conditions, that is, all devices consist of 50 nm TiO_2_ and 400 nm CdTe active layers. The current density versus voltage (*J*–*V*) characteristics of these devices under 1000 Wm^−2^ (AM 1.5 G) are shown in Figure 3a, while the dark *J*–*V* curves are presented in Figure 3b. The *J*_sc_, *V*_oc_, FF, and PCE values of the devices are summarized in Table 1. It is found that without ZnO film, the dark current is large. On the contrary, devices with a ZnO interlayer show good diode properties. The *J*_sc_, *V*_oc_, FF and PCE values of devices with the ZnO interlayer are 10.95 mA/cm^2^, 0.66 V, 30.35 and 2.22% respectively, while these values for devices without the ZnO interlayer are 7.62 mA/cm^2^, 0.63 V, 28.53, and 1.37%. We suppose that TiO_2_ deposited directly on the FTO substrate contains high interface defect density which can lead to high dark current and result in low device performance, confirming our previous work on ITO/ZnO/CdSe/CdTe/Au devices [26].

**Figure 4:**
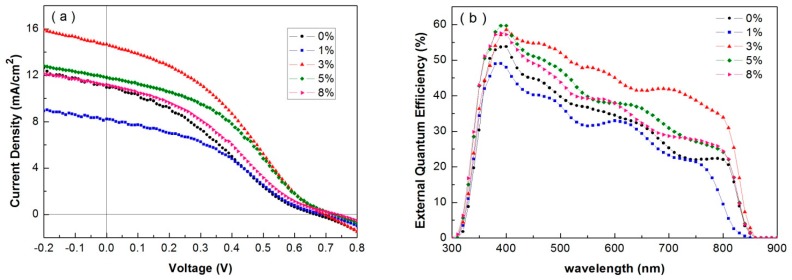
*J*–*V* characteristics of FTO/ZnO/Sb:TiO_2_/CdTe/Au with dopant concentration (**a**) under light and (**b**) EQE properties of devices.

It was reported that the conduction band edge of Sb-doped TiO_2_ is −4.22 eV, while this value is −3.81 eV for undoped TiO_2_ [30]. The conduction band edge value of Sb-doped TiO_2_ is lower than that of CdTe film (−4.0 eV), which is promising for photo electron injecting to the conduction band of TiO_2_. To investigate the effect of different Sb doping contents on device performance, we fabricated CdTe NC based solar cells with different Sb contents of 1, 3, 5, and 8% (*w*/*w*). Current density vs. voltage characteristics under AM 1.5 G illumination are shown in Figure 4a, and the device performances are listed in Table 2. The control devices (using undoped TiO_2_) shows a *V*_oc_ of 0.66 V and PCE of 2.22%, while all Sb-doped TiO_2_ devices show higher *V*_oc_. Similar device performance is obtained in the case of low Sb-doped TiO_2_ device. The PCE of device increases linearly from 1% to 3% Sb-doped content, then decreases with the further increase of Sb-doped content from 3% to 8%. The best device performance is obtained in the case of 3% (*w*/*w*) Sb-doped TiO_2_ electrodes. The *J*_sc_, *V*_oc_, FF, and PCE values of device are 14.65 mA/cm^2^, 0.70 V, 34.44, and 3.53% respectively. The PCE value of the 3% (*w*/*w*) Sb-doped device is 50 % higher than that of the control device, mainly arising from the increase in *V*_oc_ and *J*_sc_. Compared to the controlled devices, 3% or 5% Sb-doped TiO_2_ devices show higher *V*_oc_ coupled with higher PCE up to 3%. The EQE spectra (Figure 4b) show that all Sb-doped TiO_2_ devices have higher EQE almost across the entire absorbing region compared to undoped TiO_2_ devices. This suggests that the transfer of photogenerated carriers in the Sb-doped devices is more effective than that in undoped devices. It is noted that the *V*_oc_ is up to 0.69 V with different Sb-doped devices and as high as 0.74 V is obtained in the case of 8% Sb-doped device. This is, to the best our knowledge, the highest *V*_oc_ reported for solution processed CdTe NC based solar cells. However, the PCE is decreased to 2.49% for 8% Sb-doped device. We suppose that, in the case of high Sb-doped TiO_2_, excess Sb may act as recombination center for carrier, which will render the *J*_sc_ of device. It is also noted that “roll over” phenomena appear in the *J*–*V* curves for different CdTe-TiO_2_ solar cells device, which may come from large series resistance of CdTe NC film. We believe that the low FF is mainly derived from low quality of CdTe NC thin film. The compactness, morphology, and grain size of CdTe NC is greatly affected by the substrate materials even all the same parameter for processing CdTe NC film. As CdTe and CdSe NCs have similar sizes and structures (zinc-blende or wurtzite), the lattice mismatch between CdTe and CdSe is very small. High quality CdTe NC film can be obtained with CdSe NC as interlayer and high solar cells performance is expected in this case, which has been confirmed in our previously reports [25,26]. However, when CdTe NCs are deposited on FTO/ZnO/TiO_2_ by a layer-by-layer sintering process, low quality CdTe NC film with pinholes or other defects will be obtained as the lattice mismatch between CdTe and TiO_2_. The contact between CdTe and Au is non ohmic due to the large resistance existed in CdTe NC thin film, then *J*–*V* curves with cross over behavior are likely observed in this case. The principal path to CdTe NC-TiO_2_ solar cells’ further improvement lies in continued reductions in electronic trap state densities in the junction, improving the quality of CdTe NC film and optimizing the contact for CdTe NC film.

**Figure 5:**
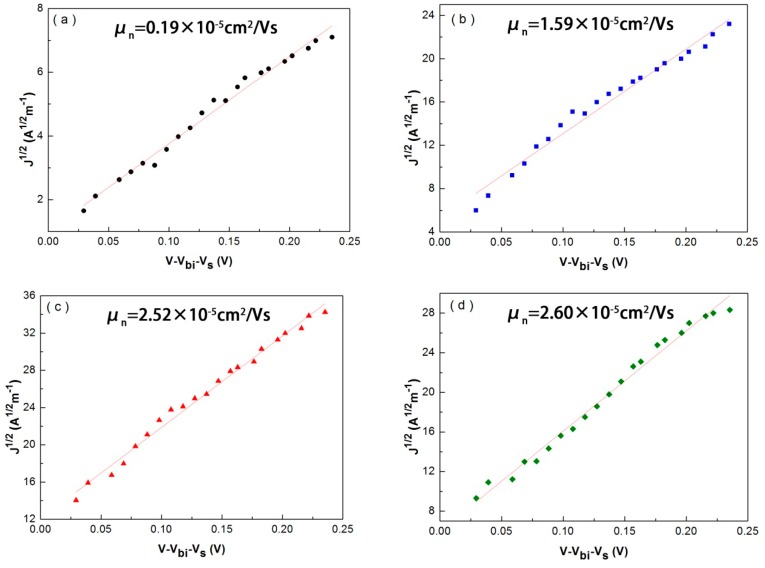
SCLC measurements of TiO_2_ thin films with dopant concentration a 0% Sb-doped (**a**); 1% Sb-doped (**b**); 3% Sb-doped (**c**); and 5% Sb-doped (**d**).

To investigate the mobility of TiO_2_ with dopant concentrations, we deposit TiO_2_ on the FTO substrate and use Al as the contact electrodes. As shown in Figure 5, the electron mobility is calculated by the formula of $J=\frac{9}{8}\frac{\varepsilon \mu_{n}V^{2}}{L^{3}}$ [13], where ε is the relative dielectric constant, *V*_bi_ is the potential difference between FTO and TiO_2_ (*V*_bi_ = 0.1 V in this case), *V*_s_ is the applied voltage, *L* is the thickness of the TiO_2_ active layer (~50 nm in this case). In the case of undoped TiO_2_, it shows a relative low electron mobility of 0.19 × 10^−^^5^ cm^2^ V^−1^ s^−1^, which implies that large surface states exist in the film. The electron mobility increases with the Sb-doped content increase linearly (as 8% and 5% Sb-doped TiO_2_ have similar mobility the *J*–*V* curve for 8% Sb-doped TiO_2_ is not presented here). The mobility of TiO_2_ film has a great effect on the performance of NC based solar cells. Film with a high mobility may markedly reduce the recombination of electrons and holes during the transfer of carriers and a high *J*_sc_ is expected to be obtained in this case, which is consistent with our *J*–*V* curve measurement of CdTe NC based solar cells with different Sb-doped TiO_2_ electrodes (see Table 2).

## 3. Materials and Methods

The preparation of TiO_2_-sol with different Sb contents was carried out under ambient conditions. In a typical 3% (*w*/*w*) Sb-doped TiO_2_ precursor, titanium *n*-butoxide (4.25 mL) was mixed with ethanolamine (3.75 mL) and ethyl alcohol (25 mL) in a beaker under continuous stirred for 2 h. 5 mL acetic acid, 5 mL deionized water, and 8.33 uL antimony(III) ethoxide (with Sb to Ti = 1:20) were added into the mixture and placed in a fume hood to allow condensation reactions. After three days, the volume of Ti-sols was about 15 mL. The Ti-sols were then transferred into a clean vial for TiO_2_ film fabrication. By varying the ratio of Sb^2+^ to titanium *n*-butoxide in the mixture, we obtained Ti-sol with dopant concentration. The fabrication of ZnO precursor and CdTe NC could be found in our previous reports [25,31].

The CdTe NC based solar cells with FTO/ZnO/TiO_2_:Sb/CdTe/Au structures were fabricated by a layer-by-layer solution process. Firstly, the ZnO precursor was spin-coated on FTO at a speed of 3000 rpm for 20 s. Then the sample was annealed at 200 °C for 10 min and then 400 °C for 10 min. Several drops of the Ti-sols were deposited on the FTO/ZnO substrates by spin coating at 2500rpm for 15 s. The sample was immediately transferred to a hot plate at 500 °C for 60 min. The deposition of CdTe film can be found in our previous report [25]. The final products were annealed at 400 °C for 15 min. Au with 80 nm thickness was deposited on the CdTe film via thermal evaporation to make back contact.

## 4. Conclusions

In conclusion, we demonstrated that inverted CdTe NC based solar cells fabricated with different Sb-doped TiO_2_ electrodes have many benefits such as simple structure, easy control, less interface defects, high electron mobility, and ideal band offsets for carrier separation, which results in good device performance. In the case of 3% Sb-doped TiO_2_ device, we obtain a high *V*_oc_ up to 0.7 V with PCE of 3.53%. *V*_oc_ as high as 0.74 V is obtained in the case of an 8% Sb-doped device. The Sb doping ratio is found to have great effect on the device performance. Our results imply that if a CdTe layer, a Sb-doped TiO_2_ layer, and their junction undergo more optimized processing (such as increasing annealing temperature or annealing time), the device performance can be further increased. This exploration gives more insight into solution processed CdTe NC based solar cells with Sb-doped TiO_2_ electrodes.

## Figures and Tables

**Table 1:** Summarized performances of CdTe-TiO_2_ heterojunction solar cells w/o ZnO film

Device Structure	*V*_oc_ (V)	*J*_sc_ (mA/cm^2^)	FF (%)	PCE (%)
FTO/TiO_2_/CdTe/Au	0.63	7.62	28.53	1.37
FTO/ZnO/TiO_2_/CdTe/Au	0.66	10.95	30.35	2.22

**Table 2:** Summarized performances of CdTe–TiO_2_ heterojunction solar cells with dopant concentration.

Sb Content (*w*/*w*)	*V*_oc_ (V)	*J*_sc_ (mA/cm^2^)	FF (%)	PCE (%)
0%	0.66	10.95	30.35	2.22
1%	0.69	8.24	34.76	1.97
3%	0.70	14.65	34.44	3.53
5%	0.72	11.83	36.58	3.13
8%	0.74	11.16	30.13	2.49
